# Exploring the comorbidity mechanisms between asthma and idiopathic pulmonary fibrosis and the pharmacological mechanisms of Bu-Shen-Yi-Qi decoction therapy via network pharmacology

**DOI:** 10.1186/s12906-022-03637-7

**Published:** 2022-06-07

**Authors:** Yuanyuan Zhong, Lingli Hu, Wenjing Chen, Bin Wang, Jing Sun, Jingcheng Dong

**Affiliations:** 1 Department of Integrative Medicine, Huashan Hospital, Fudan University, Shanghai, China; 2 Institutes of Integrative Medicine, Fudan University, Shanghai, 200040 China

**Keywords:** Asthma, IPF, BSYQ decoction, Network pharmacology, Molecule docking

## Abstract

**Backgrounds:**

Asthma and idiopathic pulmonary fibrosis (IPF) are common chronic diseases of the respiratory system in clinical practice. However, the relationship and molecular links remain unclear, and the current treatment’s efficacy is disappointing. Bu-Shen-Yi-Qi (BSYQ) decoction has proven effective in treating various chronic airway inflammatory diseases, including asthma and IPF. But the underlying pharmacological mechanisms are still to be elucidated.

**Methods:**

This study searched the proteins related to asthma and IPF via TTD, CTD, and DisGeNET databases and then submitted to the STRING to establish the protein–protein interaction (PPI) network. The co-bioinformatics analysis was conducted by Metascape. The active ingredients of BSYQ decoction were screened from TCMSP, ETCM, BATMAN-TCM databases, and HPLC/MS experiment. The corresponding targets were predicted based on TCMSP, ETCM, and BATMAN-TCM databases. The shared targets for asthma and IPF treatment were recognized, and further GO and KEGG analyses were conducted with the DAVID platform. Finally, molecule docking via Autodock Vina was employed to predict the potential binding mode between core potential compounds and targets.

**Results:**

Finally, 1333 asthma-related targets and 404 IPF-related proteins were retrieved, 120 were overlapped between them, and many of the asthma-related proteins fall into the same statistically significant GO terms with IPF. Moreover, 116 active ingredients of BSYQ decoction were acquired, and 1535 corresponding targets were retrieved. Eighty-three potential compounds and 56 potential targets were recognized for both asthma and IPF treatment. GO and KEGG analysis indicated that the inflammation response, cytokine production, leukocyte differentiation, oxygen level response, etc., were the common pathological processes in asthma and IPF, which were regulated by BSYQ decoction. Molecule docking further predicted the potential binding modes between the core potential compounds and targets.

**Conclusion:**

The current study successfully clarified the complex molecule links between asthma and IPF and found the potential common targets. Then we demonstrated the efficacy of BSYQ decoction for asthma and IPF treatment from the angle of network pharmacology, which may provide valuable references for further studies and clinical use.

**Supplementary Information:**

The online version contains supplementary material available at 10.1186/s12906-022-03637-7.

## Introduction

Asthma is one of the most common chronic non-communicable diseases, affecting about 334 million people worldwide and causing approximately 250,000 deaths [1]. It is characterized by airway hyperresponsiveness and reversible airflow restriction, with recurrent wheezing, shortness of breath, chest tightness, and cough. Idiopathic pulmonary fibrosis (IPF) is a chronic, progressive lung disease characterized by varying degrees of inflammation and fibrosis of the lung parenchyma with no definite cause [2]. The prevalence ranges from 10–60 instances per 100,000 persons, with an incidence of 2–30 cases per 100,000 person-years [3–10]. These numbers roughly equate to 130,000 persons in the United States, 300,000 in Europe, 640,000 in East Asia, and 3 million people worldwide. Both asthma and IPF are common chronic diseases of the respiratory system in clinical practice. However, the causal relationship and molecular links between them remain unclear.

Genetic susceptibility and environmental exposures are the common risk factors; persistent chronic inflammation and structural changes involving tissue remodeling and fibrosis are major features of asthma and IPF. Asthma and IPF are complex disorders resulting from risk factors and innumerate multidirectional interactions between the structural cells (bronchial epithelial cells, the alveolar epithelium fibroblasts and myofibroblasts, etc.), inflammatory cells (macrophages, neutrophils, eosinophils, and T/B lymphocytes, etc.) and extracellular matrix (ECM) whereby the relative contribution of each factor differs between individuals, different disease, and different disease subtypes. The current medications for asthma have limitations (such as glucocorticoid insensitivity, poor asthma control, side effects, etc.). Pirfenidone and nintedanib are recommended to manage IPF despite the limited efficacy in preventing disease progression and improving quality of life [11, 12]. Hence it is critical to evaluate both common pathological processes and those that are specific, recognize similarities and differences between asthma and IPF, and seek potential complementary and alternative medical treatments and strategies. Respiratory physicians search for potential novel drugs from the traditional Chinese medicine (TCM) library to treat asthma and IPF.

TCM has progressively gained wider attention worldwide due to its specific theory and long historical clinical practice [13]. Unlike modern medicine, in TCM theory, syndrome differentiation and treatment are the essential diagnosis and treatment principles for disease. TCM syndrome is a specific set of symptoms or a pattern of symptoms presenting the body's internal and external condition at a particular stage [14]. Lung-kidney deficiency is one of the prevalent clinical syndrome types in clinical practice for asthma and IPF patients, and tonify the kidney and replenishing qi is frequently-used treatment principle according to TCM theory [15, 16]. Bu-Shen-Yi-Qi formulae (BSYQ) consists of three herbs, including *Epimrdii Herba* (Yinyanghuo), *Radix Astragali* (Huangqi), and *Radix Rehmanniae* (Shengdihuang), has been demonstrated to be effective in the treatment of chronic airway inflammatory diseases based on our randomized, double-blind placebo-controlled parallel-group multicentre clinical trials [17, 18]. Experiment studies demonstrated that BSYQ decoction could relieve airway inflammation, airway hyperresponsiveness, and airway remodeling in the OVA-induced asthma mice model [19–21]. It can also reduce collagen deposition in lung tissue of bleomycin-induced pulmonary fibrosis mice model and improve pulmonary fibrosis (our unpublished data). However, it is still challenging to clarify the mechanisms of the BSYQ formula in the treatment of asthma and IPF via routine methods because TCM formulae is a complex system with multiple components, multiple targets, and synergistic interactions among its components [22].

Based on polypharmacology and systems biology, network pharmacology integrates various biological data information such as genomics, proteomics, metabolomics, and bioinformatics. It expounds on the occurrence and development of diseases from the perspective of biological network balance, understanding the interaction between the body and drugs, and guiding the rational design of drugs from the perspective of restoring or improving the balance of the biological network, which is considered to be the next-generation drug development paradigm [23–25]. At the same time, the guiding ideology of a holistic view and balance view of TCM and the overall synergistic mechanism of TCM prescription compatibility coincide with the drug research and development model advocated by network pharmacology. Therefore, integrating the emerging network pharmacology and TCM theory will provide new opportunities and methods to discover bioactive components and biomarkers, reveal their action mechanism, and explore the modern scientific connotation of TCM prescriptions based on complex biological systems [26]. Some studies have elucidated the scientific basis and systematic features of herbal medicine to treat diseases via network pharmacology, such as Xuefu Zhuyu decoction [27], Ma-huang decoction [28], Liu-Wei-Di-Huang pill [29], and Qingluoyin [30], etc.

**Fig. 1:**
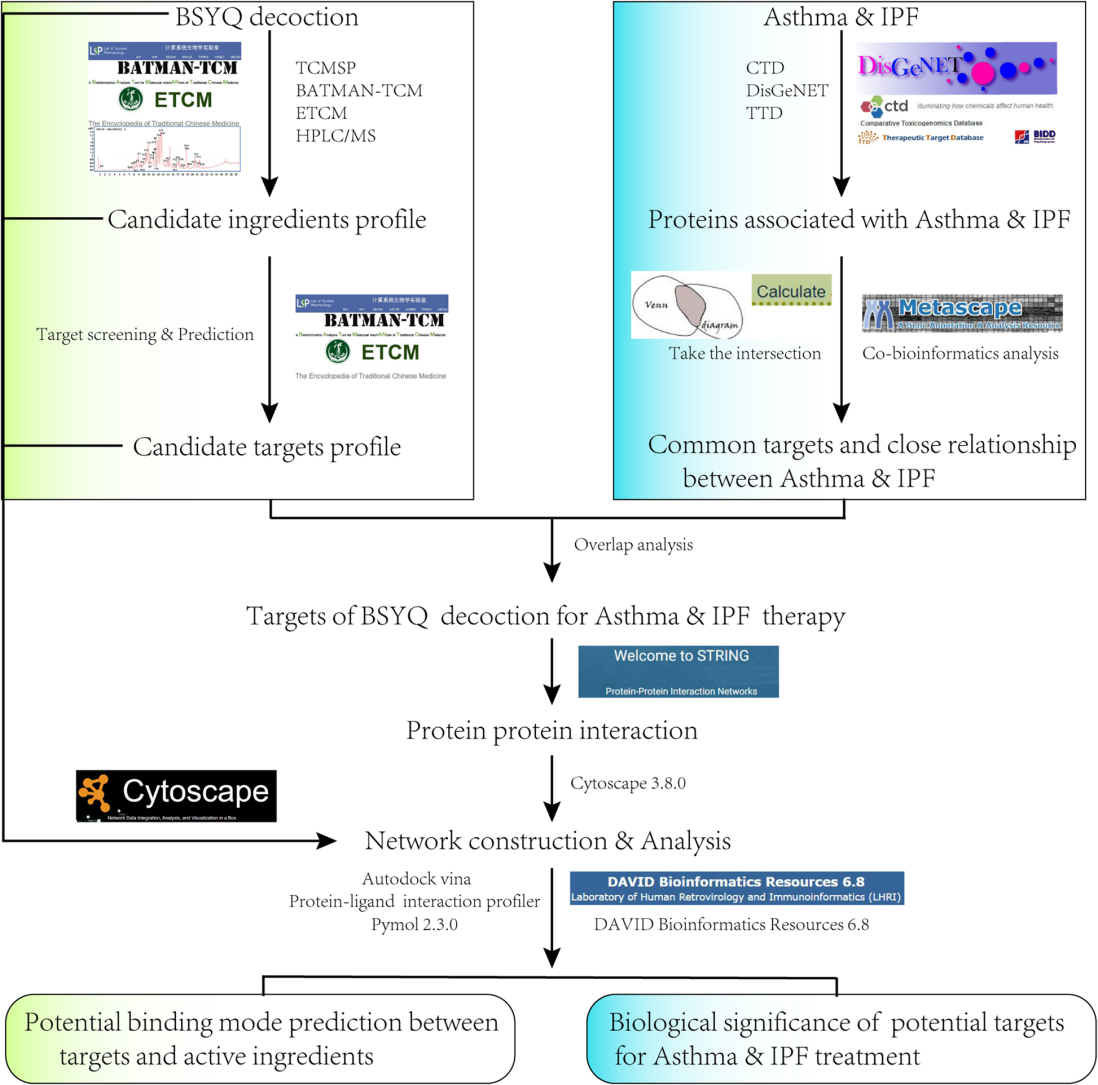
The flow chart of the current study

In the present study, we first try to explore the potential molecule links between asthma and IPF and the possible therapeutic mechanisms of BSYQ formulae for asthma and IPF and then try to understand the modern scientific connotation of the TCM theory- the same treatment for different diseases from the angle of network pharmacology (Fig. 1 depicts a flowchart of the entire research procedure).

## Materials and methods

### Asthma & IPF-related protein screening

The known target proteins for asthma and IPF were screened from the Therapeutic Target Database (TTD, http://bidd.nus.edu.sg/group/cjttd/), which is publicly accessible and provides comprehensive information about the known therapeutic protein, nucleic acid targets described in the literature, and the corresponding drugs/ligands directed at each of these targets, etc. [31]. Then we further searched the Comparative Toxicogenomics Database CTD, http://ctdbase.org/ [32] and DisGeNET database [33] (https://www.disgenet.org/) to collect the proteins related to asthma and IPF. The public CTD is an innovative digital ecosystem that connects toxicological information for chemicals, genes, phenotypes, diseases, and exposures. It now provides 45 million toxicogenomic relationships for over 16 300 chemicals, 51 300 genes, 5500 phenotypes, 7200 diseases, and 163 000 exposure events [32]. DisGeNET is a knowledge management platform integrating and standardizing data about disease-associated genes and variants from multiple sources. The latest release covers the full spectrum of human diseases (more than 24 000 diseases and traits, 17 000 genes, and 117 000 genomic variants) [33]. We searched the three databases with the keywords "asthma" or "idiopathic pulmonary fibrosis" and set the species to "Homo sapiens." Finally, we consolidated the information and removed duplicates. The common proteins of asthma and IPF were reserved for further analysis.

### Bioactive ingredients collection and targets prediction

Potential active compounds of BSYQ decoction were screened from TCMSP (http://sm.nwsuaf.edu.cn/lsp/tcmsp.php) [34], BATMAN-TCM (http://bionet.ncpsb.org/batman-tcm) [35], ETCM (http://www.nrc.ac.cn:9090/ETCM/) database [36] and the data from our previous HPLC/MS study [37]. Then the candidate targets of the active compounds were predicted based on the three databases above. TCMSP consists of all the 499 Chinese herbs registered in the Chinese pharmacopeia with 29,384 ingredients, 3,311 targets, and 837 associated diseases, as well as the ADME-related properties such as oral bioavailability (OB), half-life (HL), drug-likeness (DL), and Lipinski's rule of five (MW, AlogP, TPSA, Hdon, Hacc), etc. [34] BATMAN-TCM is the first online bioinformatics analysis tool specially designed for the research of the molecular mechanism of TCM [35]. The ETCM database includes comprehensive and standardized information for the commonly used herbs and formulas of TCM and their ingredients. It can also provide predicted target genes of TCM ingredients, herbs, and formulas, according to the chemical fingerprint similarity between TCM ingredients and known drugs [36].

### Protein–protein interaction (PPI) network construction and analysis

We took the intersection of targets of BSYQ decoction and the common proteins between asthma and IPF, then uploaded them to STRING [38] (https://string-db.org/) to generate the PPI network, the minimum required interaction score was set to high confidence (0.7) and limited to Homo sapiens The STRING database aims to collect, score and integrate all publicly available sources of protein–protein interaction information, complement these with computational predictions and then achieve a comprehensive and objective global network, including direct (physical) as well as indirect (functional) interactions [38]. The final PPI network was established and visualized via Cytoscape 3.8.0 [39]. The network parameters were calculated by NetworkAnalyzer. The MCODE app (based on vertex weighting) in Cytoscape 3.8.0 was used to search the highly connected sub-networks in the PPI network [40].

### Gene Ontology (GO) and pathway enrichment analysis

To further explore the mechanisms of BSYQ for asthma and IPF treatment, the intersection of targets of BSYQ and the common proteins between asthma and IPF were additionally performed GO enrichment and Kyoto Encyclopedia of Genes and Genomes (KEGG) pathway analysis via the online platform DAVID 6.8 [41] (DAVID, https://david.ncifcrf.gov/) and Metascape [42] (https://metascape.org/).

### Molecule docking

AutoDock vina was used in this study to evaluate the potential molecular binding mode between ingredients and candidate targets. The PyMol 2.3.0 (http://www.pymol.org/) and the online platform PLIP 2021 [43](https://plip-tool.biotec.tu-dresden.de) were employed to analyze the docked structures. The crystal structures of the target proteins were downloaded from the RCSB Protein Data Bank (www.rcsb.org). Water and hetero molecules were removed, and hydrogen atoms were added by AutoDock tools (1.5.6). The 3D chemical structures of active ingredients were retrieved from the PubChem compound database (NCBI, USA) and subjected to minimize the energy via molecular mechanics-2 (MM2) force field in Chem 3D Pro. The protein–ligand docking active site center was defined by the location of the original ligand, and the dimensions of the grids were set at 30 × 30 × 30 Ǻ in the x, y, and Z directions, with a spacing of 0.375 Ǻ between the grid points. The docked conformation corresponding to the lowest binding energy was selected as the most probable binding conformation.

## Results

### Asthma & IPF related proteins collecting and analyzing

**Fig. 2:**
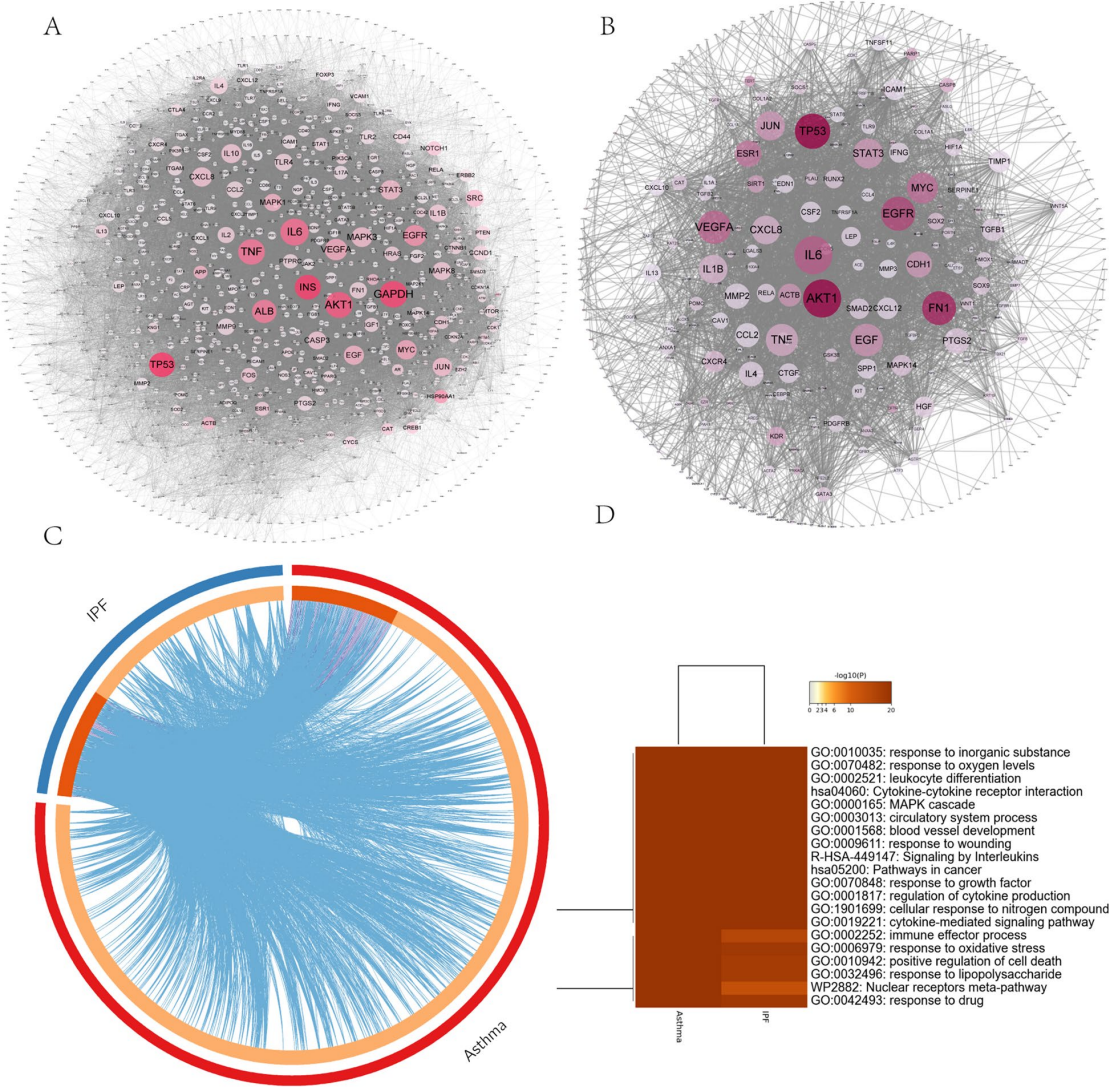
Asthma and IPF specific PPI network and co-bioinformatics analysis for the two protein profiles via Metascape. The size and color of the node are proportional to the value of the degree and betweenness centrality (**A** and **B**). **C** Circos plot for the two groups of proteins. Purple lines connect proteins that appear in both protein profiles. Blue lines connect proteins that belong to the same ontology term. **D** Top 20 common GO terms or pathways enriched by the two protein profiles

One thousand three hundred thirty-three asthma-related targets and 404 IPF-related targets were retrieved from the TTD, CTD, and DisGeNET database (Duplicates were removed and detailed in additional table S1). Asthma and IPF disease-specific PPI networks were established (Fig. 2A, B). The top 15 core proteins based on two network topology parameters (degree and betweenness centrality) in asthma and IPF were displayed in additional table S2 and S3. Then we found that VEGFA, TP53, EGFR, AKT1, EGF, IL6, STAT3, and MYC occupied the core positions in asthma and IPF-specific PPI networks, indicating the essential roles of these proteins in the pathological process of asthma and IPF. To further explore the molecule links between asthma and IPF, a co-bioinformatics analysis was conducted by Metascape. One hundred twenty proteins were overlapped in the two groups of protein lists (Fig. 2C). Much of the asthma-related proteins fall into the same statistically significant GO terms (such as response to oxygen levels, leukocyte differentiation, MAPK cascades, signaling by interleukins, response to growth factors and regulation of cytokine production, etc.) with IPF-specific proteins (Fig. 2D), indicating the strong function association between the two comparison cohorts. The 120 common proteins were used for further analysis.

### Active ingredients screening and corresponding targets prediction of BSYQ decoction

**Fig. 3:**
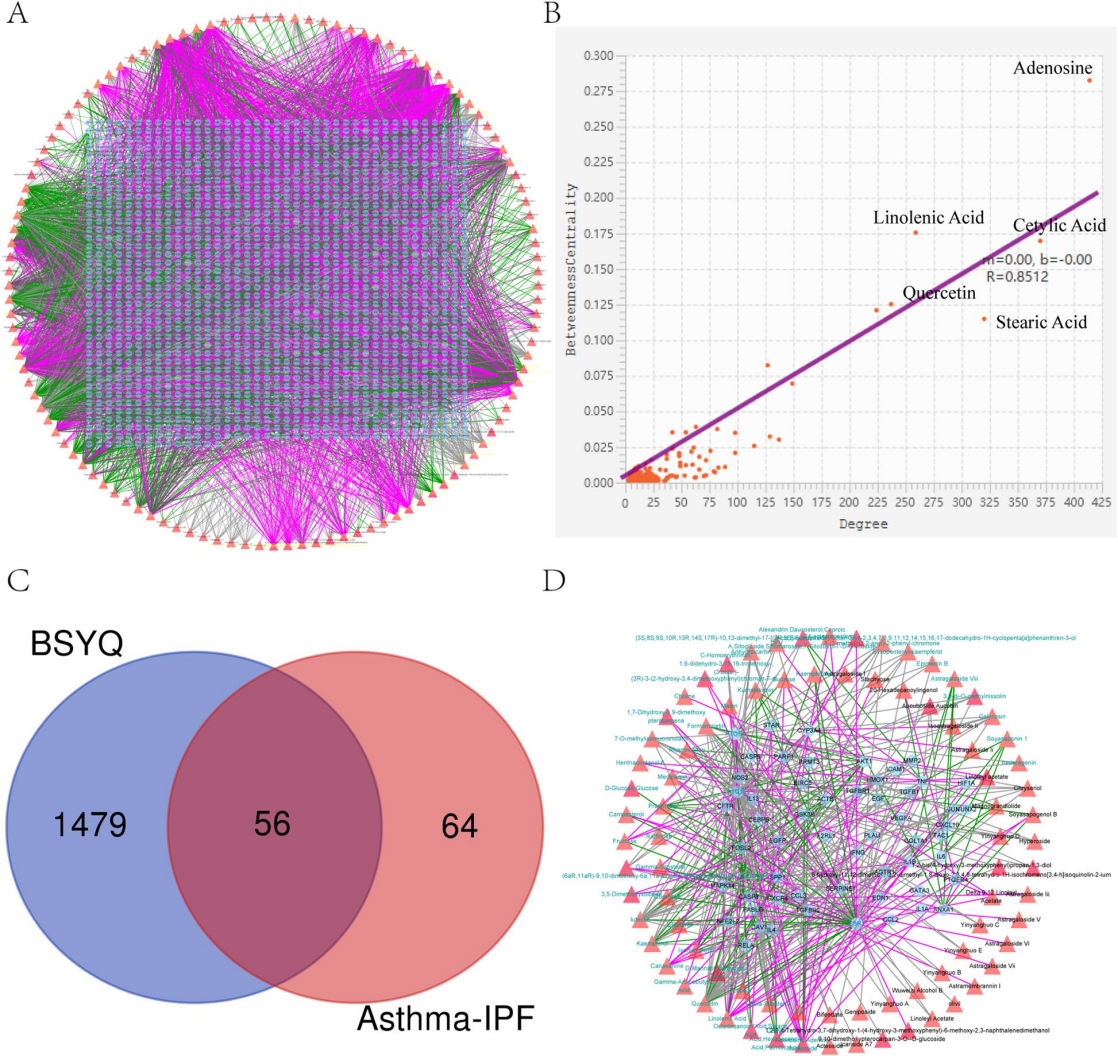
The analysis of the compounds and targets of BSYQ decoction and the potential targets screening for asthma and IPF treatment. **A** The compound-target (C-T) network, **B** the relationship between degree and betweenness centrality of the nodes in the C-T network, and the core compounds and targets of BSYQ decoction based on the two topology parameters were marked. **C** the Venn diagram between targets of BSYQ and common proteins of asthma and IPF. **D** The potential compound-potential target (PC-PT) network. The triangles and circles represent the compounds and targets, respectively

After removing duplicates, 175 active ingredients were acquired and further submitted to TCMSP, BATMAN-TCM, and ETCM databases to get the corresponding targets. Finally, except for 59 components predicted no targets, 116 active compounds, and 1535 related targets were retrieved (additional table S4 and S5). The compound-target (C-T) network was constructed and analyzed via Cytoscape 3.8.0 (Fig. 3A, B). The C-T network consists of 1651 nodes (116 active compounds and 1535 potential targets) and 5255 edges. Two centrality indicators, degree and betweenness centrality, were calculated to identify the critical nodes within the network (Fig. 3B). Interestingly, both two types of centrality indicators uniformly confirmed the core 15 candidate compounds (including adenosine, cetylic acid, octadecanoic, linolenic acid and quercetin, etc.) and targets (including PTGS2, NCOA2, AR, ESR1, and PTGS1, etc.) of BSYQ decoction ( additional table S6 and S7).

### Potential ingredients and targets of BSYQ decoction for asthma and IPF therapy

**Fig. 4:**
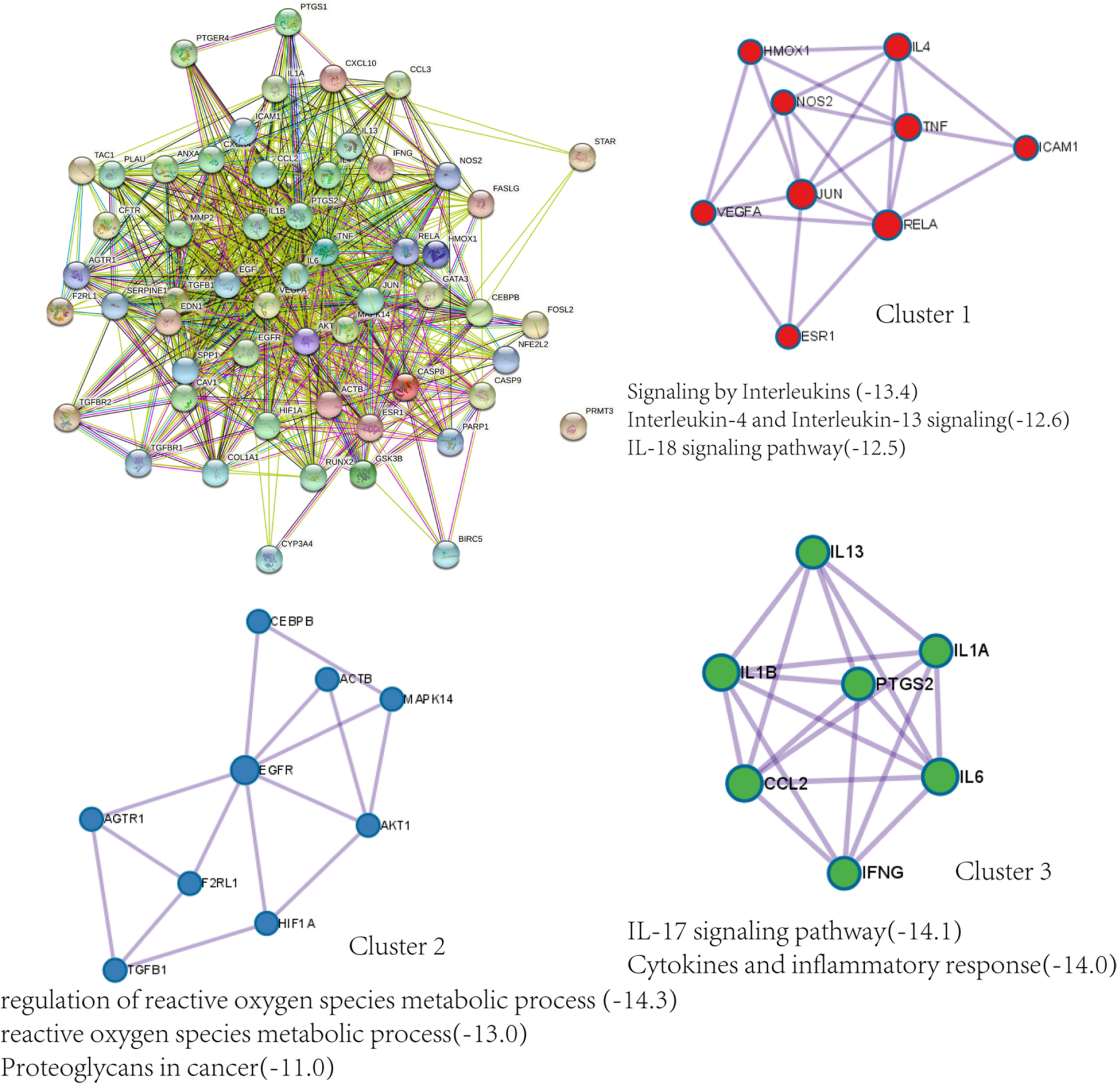
The protein–protein Interaction (PPI) network of the 56 potential targets for asthma and IPF therapy based on STRING. Similar function subnetworks were analyzed by Metascape

**Fig. 5:**
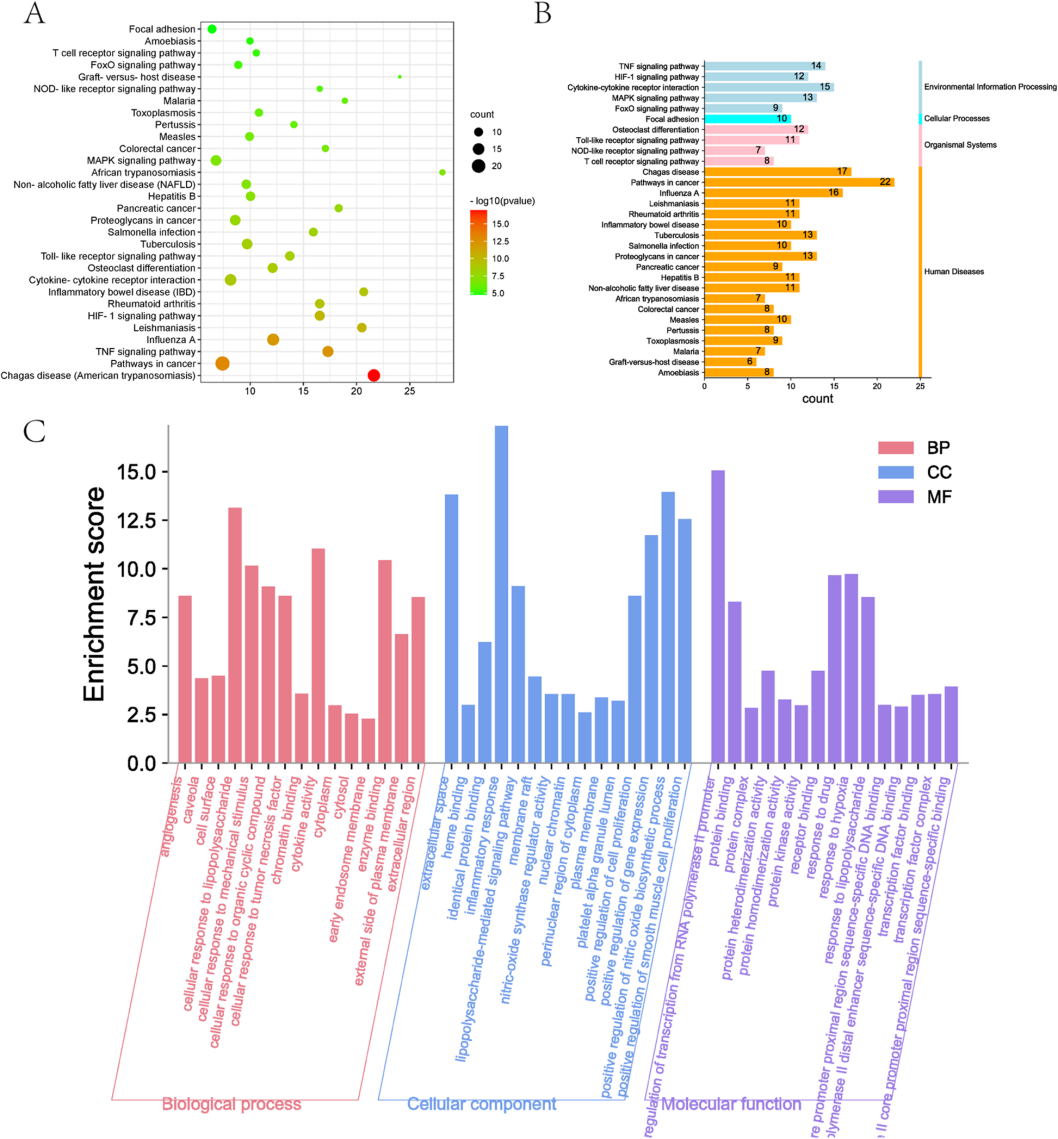
GO and KEGG analysis for the potential targets of BSYQ decoction for asthma and IPF therapy by DAVID 6.8. **A** and **B** the top 30 enriched KEGG pathways of the 56 potential targets. **C** the top 15 enriched GO items, including biology process (BP), cellular compartment (CC), and molecular function (MF)

To further explore the molecule mechanisms of BSYQ decoction for asthma and IPF therapy, we took the intersection of the targets profile of BSYQ decoction with the 120 common proteins between asthma and IPF. Finally, 56 potential targets were retrieved and were regarded as the potential targets for asthma and IPF treatment (Fig. 3C). Then a potential compound-potential target (PC-PT) network was established and analyzed (Fig. 3D). The PC-PT network consists of 139 nodes (83 potential compounds and 56 potential targets) and 371 edges. The core potential ingredients and targets based on the two network parameters are shown in Tables 1 and 2. Quercetin, luteolin, linolenic acid, adenosine, kaempferol, etc., were considered the potential core compounds, and PTGS2, ESR1, PTGS1, NOS2, AKT1, etc. were the main potential targets of BSYQ for asthma and IPF therapy. We further constructed the PPI network with the 56 potential targets by STRING and searched the similar function clusters of the PPI network by MCODE analysis based on topology (Fig. 4). The top 15 core proteins based on the two topological parameters in the 56 potential targets PPI network are shown in Table 3. IL6, IL-1β, TNF, VEGFA, AKT1, etc., played an essential role in the PPI network, indicating the crucial roles in treating asthma and IPF. Similar function subnetworks were constructed, and function analysis showed that cluster 1 mainly participated in the interleukins signaling (Fig. 4B). Cluster 2 specifically regulates the reactive oxygen species (Fig. 4C). Cluster 3 mainly regulates the cytokines and inflammatory response (Fig. 4D). Then we performed the GO and KEGG analysis with the 56 potential targets (Fig. 5). KEGG pathway analysis showed that TNF signaling pathway, HIF-1 signaling pathway, cytokine-cytokine receptor interaction, toll-like receptor signaling pathway, MAPK signaling pathway, etc. were enriched and regulated by BSYQ decoction (Fig. 5A, 5B), indicating the underline comprehensive mechanisms of BSYQ decoction for asthma and IPF treatment. We found that the 56 potential targets mainly participate in the regulation of the inflammatory response, nitric oxide biosynthetic process, smooth muscle cell proliferation process, etc. (Fig. 5C).

**Table 1:** Top 15 potential compounds in the PC-PT network according to degree and betweenness centrality

Ingredient Name	Degree	Betweenness Centrality	PubChem CID	Source	Ingredient Name	Degree	Betweenness Centrality	PubChem CID	Source
Quercetin	36	0.309833105	5,280,343	BATMAN-TCM, TCMSP	Quercetin	36	0.309833105	5,280,343	BATMAN-TCM, TCMSP
luteolin	20	0.074877417	5,280,445	TCMSP	Sucrose	6	0.104421054	5988	BATMAN-TCM, ETCM
Linolenic Acid	16	0.076791716	5,280,934	ETCM	Adenosine,Adenine Nucleoside	15	0.079102825	60,961	ETCM
Adenosine,Adenine Nucleoside	15	0.079102825	60,961	ETCM	Linolenic Acid	16	0.076791716	5,280,934	ETCM
Kaempferol	14	0.048065771	5,280,863	BATMAN-TCM, TCMSP	luteolin	20	0.074877417	5,280,445	TCMSP
Isorhamnetin	11	0.010756097	5,281,654	BATMAN-TCM, TCMSP	Kaempferol	14	0.048065771	5,280,863	BATMAN-TCM, TCMSP
Rhamnocitrin	10	0.00855788	5,320,946	BATMAN-TCM	Canavanine	6	0.033126673	439,202	BATMAN-TCM
Pratensein	10	0.00855788	5,281,803	HPLC/MS	FA	6	0.029886082	6037	TCMSP
Formononetin	10	0.01317824	5,280,378	BATMAN-TCM, HPLC/MS, TCMSP	Fructose	4	0.019612123	5984	ETCM
Beta-Sitosterol	9	0.019445351	222,284	BATMAN-TCM	Beta-Sitosterol	9	0.019445351	222,284	BATMAN-TCM
Cetylic Acid,Hexadecanoic Acid,Palmitic Acid	8	0.017682546	985	ETCM	Cetylic Acid,Hexadecanoic Acid,Palmitic Acid	8	0.017682546	985	ETCM
Kumatakenin	7	0.005434062	5,318,869	BATMAN-TCM, ETCM, TCMSP	Hentriacontanol-6	2	0.014492754	5,318,007	BATMAN-TCM
Canavanine	6	0.033126673	439,202	BATMAN-TCM	Medicarpin	6	0.013850015	336,327	ETCM
Sucrose	6	0.104421054	5988	BATMAN-TCM, ETCM	(6aR,11aR)-9,10-dimethoxy-6a,11a-dihydro-6H-benzofurano[3,2-c]chromen-3-ol	6	0.013850015	14,077,830	TCMSP
Octadecanoic?Acid,Stearic Acid	6	0.010597854	5281	ETCM	Formononetin	10	0.01317824	5,280,378	BATMAN-TCM, HPLC/MS, TCMSP

**Table 2:** Top 15 potential targets in the PC-PT network according to degree and betweenness centrality

Protein Name	Degree	Betweenness Centrality	Protein Name	Degree	Betweenness Centrality
PTGS2	54	0.277312173	PTGS2	54	0.277312173
ESR1	46	0.153120648	PTGS1	42	0.204507204
PTGS1	42	0.204507204	ESR1	46	0.153120648
NOS2	19	0.032615148	CXCR4	8	0.086469588
AKT1	15	0.01512124	TNF	12	0.060803175
GSK3B	13	0.009859331	CYP3A4	12	0.058290209
CYP3A4	12	0.058290209	HMOX1	6	0.035902065
TNF	12	0.060803175	HIF1A	4	0.035245261
ACTB	10	0.013813159	ICAM1	5	0.034843635
MAPK14	10	0.001209612	NOS2	19	0.032615148
CEBPB	9	0.007230542	IL1B	8	0.031406599
ANXA1	8	0.014493758	TGFBR2	4	0.029023858
CXCR4	8	0.086469588	IFNG	4	0.016903104
IL1B	8	0.031406599	AKT1	15	0.01512124
IL6	6	0.009704189	SERPINE1	5	0.014896122

**Table 3:** Top 15 potential targets in the 56 potential targets PPI network

Protein Name	Degree	Betweenness Centrality	Protein Name	Degree	Betweenness Centrality
IL6	51	0.059767121	IL6	51	0.059767121
IL1B	46	0.034930701	EGFR	43	0.041706178
TNF	46	0.030700549	VEGFA	46	0.038701872
VEGFA	46	0.038701872	IL1B	46	0.034930701
AKT1	45	0.034171523	AKT1	45	0.034171523
PTGS2	44	0.027625229	TNF	46	0.030700549
EGFR	43	0.041706178	ESR1	33	0.030204633
EGF	42	0.022481519	PTGS2	44	0.027625229
JUN	40	0.025683985	JUN	40	0.025683985
CCL2	39	0.015488887	EGF	42	0.022481519
MMP2	37	0.009780132	MAPK14	34	0.021812819
IL4	36	0.013415867	CCL2	39	0.015488887
TGFB1	35	0.009526729	IL4	36	0.013415867
MAPK14	34	0.021812819	CEBPB	20	0.011576047
ICAM1	33	0.005648341	CXCL10	27	0.011252843

### Molecule docking for the core potential ingredients and targets of BSYQ for asthma and IPF treatment

**Fig. 6:**
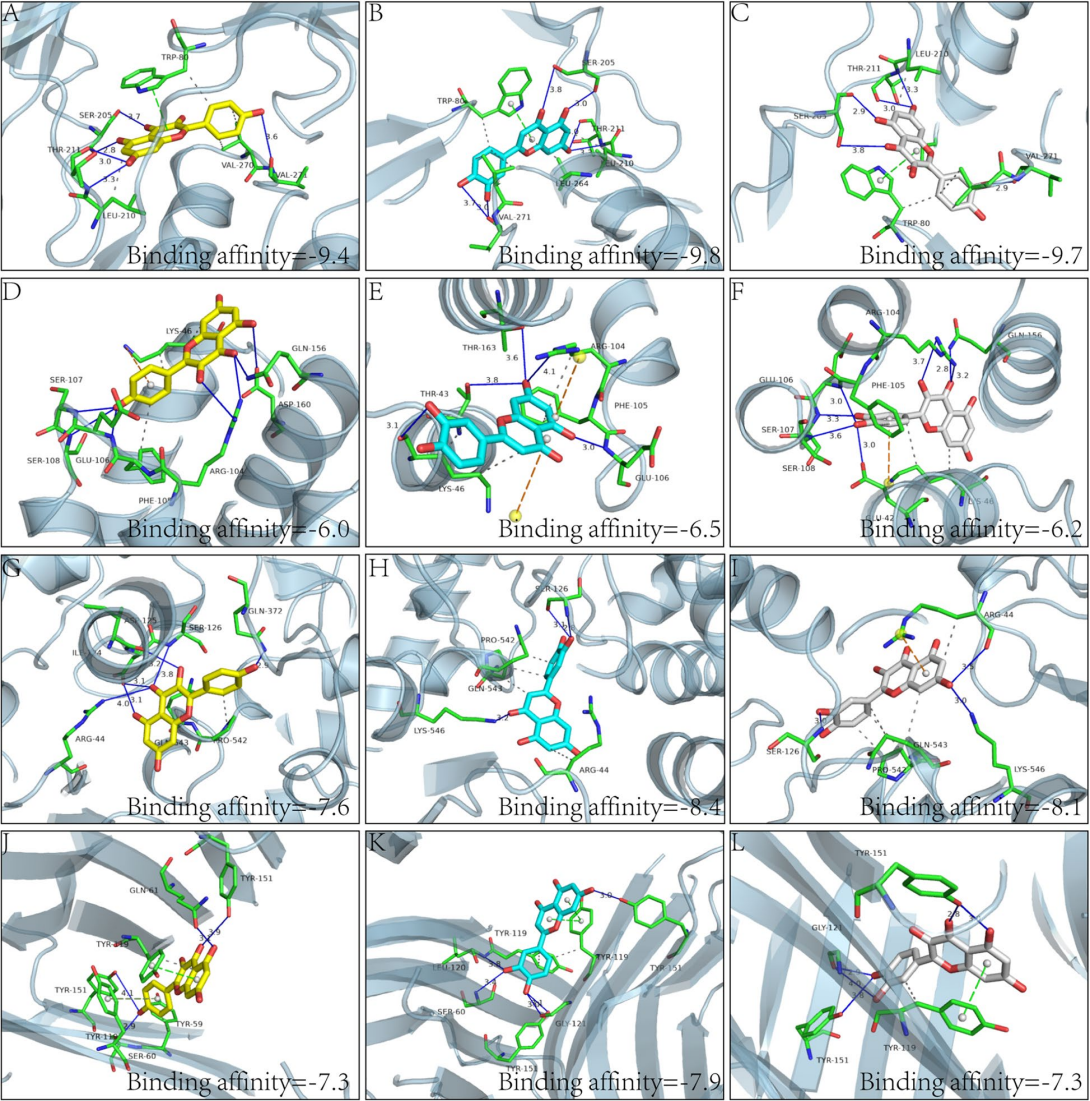
Predicted binding mode within the active site of the drug-target complexes obtained from Autodock vina. **A** kaempferol-AKT1; **B** luteolin-AKT1; **C** quercetin-AKT1; **D** kaempferol-IL6; **E** luteolin-IL6; **F** quercetin-IL6; **G** kaempferol-PTGS2; **H** luteolin-PTGS2; **I** quercetin-PTGS2; **J** kaempferol-TNF; **K** luteolin-TNF; **L**-quercetin-TNF). The proteins were presented as cartoon modes, and molecules were presented as ball and stick models. Active site amino acid residues are represented as lines. Dotted blue lines in these pictures represent hydrogen bonds with distance units of ˚ A, dotted khaki lines represent π-Cation interactions, dotted red lines represent π-Stacking (parallel) interactions and dotted grey lines represent hydrophobic interaction. Other O and N atoms are colored red and blue, respectively

In the current study, the possible interaction modes between core ingredients and targets were predicted by Autodock vina. Molecule docking is a computational method that efficiently predicts the noncovalent binding of macromolecules or, more frequently, of a macromolecule (receptor) and a small molecule (ligand). It is generally believed that the lower the binding energy between ligand and receptor, the greater the possibility of interaction. Three core ingredients, including quercetin, luteolin, and kaempferol with four corresponding essential targets including AKT1, IL-6, PTGS2, and TNF, were docked and displayed to elucidate the exact binding mode (Fig. 6, A: kaempferol-AKT1; B: luteolin-AKT1; C: quercetin-AKT1; D: kaempferol-IL6; E: luteolin-IL6; F: quercetin-IL6; G: kaempferol-PTGS2; H: luteolin-PTGS2; I: quercetin-PTGS2; J: kaempferol-TNF; K: luteolin-TNF; L: quercetin-TNF). Specifically, taking the kaempferol with AKT1, for example, kaempferol was located within the interfaced pocket formed by active amino acid residues of AKT1, five conventional hydrogen bonds were formed between kaempferol and AKT1 by interacting with the vital amino acids, including SER205, THR211, and VAL271. Additionally, π-stacking between kaempferol and TRP80, hydrophobic interactions with TRP80, LEU210, and VAL270 were found in the active site, which helped stabilize the compound at the binding site (Fig. 6A). Six key hydrogen bonds with SER205A, THR211A, and VAL271A, hydrophobic interactions with TRP80A, LEU210A, LEU240A, and VAL270A, and π-Stacking interaction with TRP80A, were established between luteolin and AKT1 (Fig. 6B). Similarly, quercetin and AKT1 were shown to create five critical hydrogen bonds with SER205A, THR211A, and VAL271A, hydrophobic contacts with TRP80A, LEU210A, VAL270A, and ASP292A, and π-Stacking interaction with TRP80A (Fig. 6C). Between kaempferol and IL-6, seven important hydrogen bonds were discovered with ARG104A, GLU106A, SER108A, GLN156A, and ASP160A, as well as hydrophobic interactions with LYS46A and PHE105A, and π-Cation interactions with LYS46A were found (Fig. 6D). Five critical hydrogen bonds with THR43A, LYS46A, ARG 104A, GLU106A, and THR163A, hydrophobic interactions with LYS46A, ARG104A, and PHE105A, and π-Cation interactions with LYS46A and ARG 104A were formed between luteolin and IL-6 (Fig. 6E). Quercetin and IL-6 formed seven critical hydrogen bonds with GLU42A, ARG104A, GLU106A, SER107A, SER108A, and GLN156A, as well as hydrophobic interactions with LYS46A and PHE105A, and π-Cation interactions with LYS46A (Fig. 6F). Between kaempferol and PTGS2, six critical hydrogen bonds with ARG44A, ILE124A, ASP125A, SER126A, and GLN372A, as well as hydrophobic interactions with PRO542B and GLN543B, were discovered (Fig. 6G). Three key hydrogen bonds with SER126A and LYS546B, hydrophobic interactions with ARG44A, PRO542B, and GLN543B, were established between luteolin and PTGS2 (Fig. 6H). Quercetin and PTGS2 were shown to have three critical hydrogen bonds with ARG44A, SER126A, and LYS546B. Hydrophobic interactions with ARG44A, PRO542B, and GLN543B, and π-Cation interaction with ARG44A were predicted (Fig. 6I). Between kaempferol and TNF, four key hydrogen bonds with SER60B, GLN61A, TYR151A, and TYR151B, hydrophobic interactions with TYR59B and TYR119A, and π-Stacking interaction with TYR119A and TYR119B, were recognized (Fig. 6J). Five key hydrogen bonds with SER60B, LEU120B, GLY121A, TYR151A, and TYR151B, hydrophobic interactions with TYR59B and TYR119A, and π-Stacking interaction with TYR119A and TYR119B, were formed between luteolin and TNF (Fig. 6K). Quercetin and TNF established five critical hydrogen bonds with GLY121A, TYR151A, and TYR151B, hydrophobic interactions with TYR119A, and π-Stacking interactions with TYR119A (Fig. 6L). Taken together, hydrogen-bonding, π-stacking, π-cation, and hydrophobic interactions played key roles in the protein − ligand recognition and stability, which may be helpful for the activation or inhibition of the target proteins and is necessary for the pharmacology activities.

## Discussion

Both asthma and IPF are inflammatory lung diseases characterized by airway injury, inflammation, bronchial and parenchymal remodeling [44]. The pathogenesis of asthma has not been fully defined, involving immunology, neuroendocrinology, genetic factors, and environmental factors. Airway hyperresponsiveness and airflow restriction are the main pathological features, and chronic inflammation is the main trigger. IPF is now generally considered the result of the interactions of multiple genetic and environmental risk factors. The aging alveolar epithelial repetitive local micro-injuries trigger abnormal epithelial fibroblast communication, induce myofibroblasts and extracellular matrix accumulation, and pulmonary interstitial remodeling [45]. Chronic airway inflammation, epithelial-mesenchymal transformation (EMT), and oxidative stress also participate in the occurrence and development of IPF [46, 47]. Thus, the repeated airway epithelial injury, chronic airway inflammation, EMT, airway remolding, and their interactions play essential roles in the pathological process in asthma and IPF, indicating the similarity between the two diseases. Unfortunately, the anti-inflammatory therapy for asthma can only control symptoms, and the improvement of disease progression is limited. What's more, it did not improve the outcome in the treatment of IPF, and an immunosuppressive therapeutic strategy incorporating prednisolone and azathioprine was shown to increase mortality [48, 49]. The nintedanib and pirfenidone cannot completely prevent the progressive decline of pulmonary function [50]. So, seeking new alternative therapies for IPF and asthma treatment is highly urgent and of far-reaching significance.

TCM is a comprehensive medicinal system that has been used in clinical practice for thousands of years and plays a vital role in the health maintenance of people all over the world [51, 52]. The validated curative effects of TCM make it a feasible alternative therapeutic agent for disease treatment [27]. Then, BSYQ decoction, proven effective, is regarded as the ideal joint therapy for asthma and IPF. The current study first explored the complex molecule links between asthma and IPF. We constructed asthma and IPF-specific PPI networks and compared the two protein profiles. The co-bioinformatic analysis showed that inflammation response, cytokine production, leukocyte differentiation, oxygen level response, etc., commonly participate in the progress of asthma and IPF and found 120 proteins overlapped. Additionally, eight proteins, including VEGFA, TP53, EGFR, AKT1, EGF, IL6, STAT3, and MYC, played essential roles in asthma and IPF. Then we searched the active compounds and predicted the corresponding targets based on the TCMSP, BATMAN-TCM, and ETCM databases. Finally, 175 active compounds (with 59 no predicted targets) and 1535 predicted targets were acquired. Then 83 potential targets anchored 56 common proteins between asthma and IPF, and the core potential compounds and targets were recognized. The additional GO and KEGG analysis indicated that inflammatory response, nitric oxide biosynthetic process, smooth muscle cell proliferation, etc., were mainly regulated by BSYQ decoction both in asthma and IPF. We also constructed the PPI network based on the STRING database, searched the similar function clusters (the interleukins signaling, oxygen species metabolism, and the cytokines and inflammatory response), and further verified the potential binding mode between the potential compounds and targets via the molecule docking method. Unlike modern medicine anchored single targets, BSYQ decoction consists of 83 potential compounds and targets 56 common targets of asthma and IPF, regulated several pathways and biological processes, and showed a synthetic therapeutic effect.

IL-6, TNF, and AKT, which occupied an important position in asthma and IPF, are essential targets regulated by BSYQ decoction. IL-6 binds to sIL-6R and activates the membrane-bound glycoprotein 130 (gp130), then actives Jak/signal transducer and activator of transcription (STAT) signaling pathway [53], which is implicated in a variety of inflammatory processes, including IPF [54, 55] and asthma [56]. Increased levels of tumor necrosis factor (TNF) α have been linked to several pulmonary inflammatory diseases, including asthma, chronic obstructive pulmonary disease (COPD), acute lung injury (ALI), acute respiratory distress syndrome (ARDS), sarcoidosis, and IPF. TNF-α plays multiple roles in disease pathology by inducing an accumulation of inflammatory cells, stimulating the generation of inflammatory mediators, and causing oxidative and nitrosative stress, airway hyperresponsiveness, and tissue remodeling [44]. AKT regulates many processes, including metabolism, proliferation, cell survival, growth, and angiogenesis, and targeting the PI3K/AKT signal pathway effectively treats asthma and IPF [57, 58]. In the present study, we found that IL-6 was anchored by 6 potential ingredients of BSYQ decoction (astragaloside Viii, magnograndiolide, soyasaponin 1, luteolin, quercetin, and aucuboside), TNF was targeted by 12 potential ingredients (adenosine, linolenic acid, sucrose, alexandrin, astragaloside Viii, magnograndiolide, soyasaponin 1, kaempferol, luteolin, quercetin, aucuboside, and cetylic acid) and AKT was hit by 11 ingredients (adenosine, DFV, formononetin, isorhamnetin, kaempferol, kumatakenin, luteolin, patensein, quercetin, quercitrin, and rhamnocitrin). Multi compounds anchored IL-6, TNF, and AKT and then produced synergistic effects. Representative flavonoids, including quercetin, kaempferol, and luteolin, were regarded as the core compounds for asthma and IPF treatment of BSYQ prescription based on our study. The anti-inflammatory and immunomodulating properties of quercetin are effectively utilized in the treatment of late-phase, and late-late-phase bronchial asthma responses, which is more competent in inhibiting IL-8 than cromolyn [59]. It can regulate the Th1/Th2 stability and decrease the antigen-specific IgE antibody released by B cells [60]. At the same time, it can reverse bleomycin-induced pulmonary fibrosis and attenuate lethality, weight loss, and the expression of pulmonary senescence markers by promoting FasL receptor and caveolin-1 expression and inhibiting AKT activation [61]. Kaempferol is a flavonoid found in many edible plants. Its anti-oxidant/anti-inflammatory effects have been demonstrated in disease models such as diabetes and asthma [62]. It can alleviate airway inflammation by modulating the Tyk2-STAT1/3 signaling response in the endotoxin-exposed airway epithelium in asthmatic mice [63]. But the efficacy of IPF treatment has not been evaluated. We found that it may be a potential agent for IPF therapy for the first time. Luteolin can modulate OVA-induced airway bronchoconstriction and bronchial hyperreactivity [64], inhibit autophagy by activating PI3K/Akt/mTOR signaling and inhibit the Beclin-1-PI3KC3 complex [65]. It can reduce the weight index and hydroxyproline content, delay the process of pulmonary fibrosis and inhibit TGF-β1 mRNA expression in the bleomycin-induced pulmonary fibrosis model [66, 67]. Particularly, IL-13 (anchored by Linolenic Acid) is a key Th2 cytokine that induces airway inflammation and remodeling. It is recognized as a central mediator of allergic asthma [68, 69]. The anti-interleukin-4 receptor α monoclonal antibody (Dupilumab) that blocks both interleukin-4 and interleukin-13 signaling significantly decreased the rates of severe asthma exacerbation, as well as improved lung function and better asthma control [70]. It's also a stimulator of fibroblast proliferation and extracellular matrix synthesis in the process of IPF [71]. IL-13 and its receptors are elevated in IPF bronchoalveolar lavage fluid [72], while neutralization of IL-13 attenuated bleomycin-induced pulmonary fibrosis [73]. In summary, multiple active ingredients in BSYQ decoction can act on various targets to treat diseases and then play a synthetic therapeutic effect.

Despite the profound significance of this study, several limitations should be noted. Firstly, the network construction and analysis separated from biological entities cannot fully reflect the internal network regulation mechanisms and dynamic changes of disease. Secondly, there is a dose–effect relationship between drugs and diseases, and the current network pharmacology method is challenging to achieve the purpose of quantification.

## Conclusion

The relationship between asthma and IPF is complicated, and clinical and experimental studies have proved the efficacy of BSYQ decoction for treating asthma and IPF. The current study successfully clarified the complex molecule links between asthma and IPF and found the potential common targets. Then we demonstrated the efficacy of BSYQ decoction for asthma and IPF treatment from the angle of network pharmacology, which may provide valuable references for further studies and clinical use.

## Supplementary Information

**Additional file 1: Supplementary Table S1. **Proteins related to asthma and IPF based on TTD, CTD and DisGeNET database.

**Additional file 2: Table S2.** Top 15 core proteins in the asthma-specific PPI network. **Table S3.** Top 15 core proteins in the IPF specific PPI network.

**Additional file 3: Supplementary Table S4. **Candidate compounds of BSYQ decoction.

**Additional file 4: Supplementary Table S5. **Candidate compunds and predicted targets based on TCMSP, ETCM and BATMAN-TCM.

**Additional file 5: Table S6.** Top 15 active compounds in the C-T network according to degree and betweenness centrality. **Table S7.** Top 15 candidate targets in the C-T network according to degree and betweenness centrality.

## Data Availability

The datasets used and/or analyzed during the current study are available from the corresponding author on reasonable request.

## Abbreviations

IPF: Idiopathic pulmonary fibrosis
BSYQ decoction: Bu-Shen-Yi-Qi decoction
TTD: Therapeutic Target Database
CTD: Comparative Toxicogenomics Database
TCMSP: Traditional Chinese Medicine Systems Pharmacology Database and Analysis Platform
BATMAN-TCM: A Bioinformatics Analysis Tools for Molecular mechanism of Traditional Chinese Medicine
ETCM: The Encyclopedia of Traditional Chinese Medicine
TCM: Traditional Chinese Medicine
PPI: Protein–protein interaction
DAVID: The Database for Annotation, Visualization, and Integrated Discovery
AKT1: RAC-alpha serine/threonine-protein kinase
IL-6: Interleukin-6
PTGS2: Prostaglandin G/H synthase 2
TNF: Tumor necrosis factor
VEGFA: Vascular endothelial growth factor
TP53: Cellular tumor antigen p53
EGFR: Epidermal growth factor receptor
EGF: Pro-epidermal growth factor
STAT3: Signal transducer and activator of transcription 3
MYC: Myc proto-oncogene protein
