# Supplementary material for: Exploring the comorbidity mechanisms between asthma and idiopathic pulmonary fibrosis and the pharmacological mechanisms of Bu-Shen-Yi-Qi decoction therapy via network pharmacology
Source: BMC Complement Med Ther. 2022 Jun 7;22:151. doi: 10.1186/s12906-022-03637-7 (PMC9175349; doi:10.1186/s12906-022-03637-7)
Supplement: Supplementary file 2 — Additional file 2: Table S2. Top 15 core proteins in the asthma-specific PPI network. Table S3. Top 15 core proteins in the IPF specific PPI network. [file 12906_2022_3637_MOESM2_ESM.docx]

**Table S2** Top 15 core proteins in the asthma-specific PPI network.

| Protein Name | Degree | Betweenness Centrality | Protein Name | Degree | Betweenness Centrality |
| --- | --- | --- | --- | --- | --- |
| IL6 | 507 | 0.026602 | TP53 | 463 | 0.038446 |
| GAPDH | 500 | 0.035006 | INS | 464 | 0.03671 |
| AKT1 | 490 | 0.031011 | GAPDH | 500 | 0.035006 |
| TNF | 479 | 0.025118 | AKT1 | 490 | 0.031011 |
| INS | 464 | 0.03671 | IL6 | 507 | 0.026602 |
| TP53 | 463 | 0.038446 | TNF | 479 | 0.025118 |
| ALB | 434 | 0.023965 | ALB | 434 | 0.023965 |
| VEGFA | 400 | 0.014278 | EGFR | 391 | 0.01915 |
| EGFR | 391 | 0.01915 | HSP90AA1 | 243 | 0.019129 |
| MAPK3 | 367 | 0.010079 | MYC | 360 | 0.014939 |
| STAT3 | 362 | 0.010978 | VEGFA | 400 | 0.014278 |
| MYC | 360 | 0.014939 | NME8 | 54 | 0.012606 |
| CXCL8 | 358 | 0.008725 | EGF | 354 | 0.012404 |
| EGF | 354 | 0.012404 | APP | 236 | 0.012177 |
| IL10 | 353 | 0.008187 | MAPK1 | 316 | 0.012167 |

**Table S3** Top 15 core proteins in the IPF specific PPI network.

| Protein Name | Degree | Betweenness Centrality | Protein Name | Degree | Betweenness Centrality |
| --- | --- | --- | --- | --- | --- |
| IL6 | 129 | 0.049704 | TP53 | 119 | 0.083659 |
| AKT1 | 129 | 0.082012 | AKT1 | 129 | 0.082012 |
| TP53 | 119 | 0.083659 | FN1 | 114 | 0.070219 |
| EGFR | 115 | 0.05726 | EGFR | 115 | 0.05726 |
| FN1 | 114 | 0.070219 | IL6 | 129 | 0.049704 |
| VEGFA | 112 | 0.046531 | VEGFA | 112 | 0.046531 |
| EGF | 110 | 0.029447 | MYC | 104 | 0.042975 |
| TNF | 108 | 0.020339 | ESR1 | 88 | 0.039777 |
| MYC | 104 | 0.042975 | EGF | 110 | 0.029447 |
| STAT3 | 100 | 0.026734 | TERT | 34 | 0.02814 |
| JUN | 98 | 0.027703 | JUN | 98 | 0.027703 |
| IL1B | 96 | 0.016969 | ACTB | 76 | 0.027551 |
| CXCL8 | 96 | 0.016948 | STAT3 | 100 | 0.026734 |
| ESR1 | 88 | 0.039777 | CDH1 | 87 | 0.026659 |
| CDH1 | 87 | 0.026659 | CFTR | 28 | 0.025087 |
