# Supplementary material for: Exploring the comorbidity mechanisms between asthma and idiopathic pulmonary fibrosis and the pharmacological mechanisms of Bu-Shen-Yi-Qi decoction therapy via network pharmacology
Source: BMC Complement Med Ther. 2022 Jun 7;22:151. doi: 10.1186/s12906-022-03637-7 (PMC9175349; doi:10.1186/s12906-022-03637-7)
Supplement: Supplementary file 5 — Additional file 5: Table S6. Top 15 active compounds in the C-T network according to degree and betweenness centrality. Table S7. Top 15 candidate targets in the C-T network according to degree and betweenness centrality. [file 12906_2022_3637_MOESM5_ESM.docx]

**Table S6** Top 15 active compounds in the C-T network according to degree and betweenness centrality.

| Ingredient Name | Degree | Betweenness Centrality | Ingredient Name | Degree | Betweenness Centrality |
| --- | --- | --- | --- | --- | --- |
| Adenosine, Adenine Nucleoside | 414 | 0.282464182 | Adenosine, Adenine Nucleoside | 414 | 0.282464 |
| Cetylic Acid,Hexadecanoic Acid,Palmitic Acid | 370 | 0.169742477 | Linolenic Acid | 259 | 0.175644 |
| Octadecanoic?Acid,Stearic Acid | 320 | 0.114930316 | Cetylic Acid,Hexadecanoic Acid,Palmitic Acid | 370 | 0.169742 |
| Linolenic Acid | 259 | 0.175644195 | Quercetin | 237 | 0.125366 |
| Quercetin | 237 | 0.125365848 | Gamma-Aminobutyric Acid | 224 | 0.121065 |
| Gamma-Aminobutyric Acid | 224 | 0.121065221 | Octadecanoic?Acid,Stearic Acid | 320 | 0.11493 |
| Canavanine | 149 | 0.069555043 | FA | 127 | 0.082434 |
| Kaempferol | 137 | 0.030226515 | Canavanine | 149 | 0.069555 |
| luteolin | 129 | 0.032355609 | Sucrose | 63 | 0.039162 |
| FA | 127 | 0.082434046 | Uridine | 82 | 0.037645 |
| Beta-Sitosterol | 115 | 0.02578383 | D-Mannitol,Cordycepic Acid | 98 | 0.034955 |
| D-Mannitol,Cordycepic Acid | 98 | 0.034955394 | luteolin | 129 | 0.032356 |
| Lupeol | 98 | 0.020896911 | Kaempferol | 137 | 0.030227 |
| Isorhamnetin | 89 | 0.011006298 | Beta-Sitosterol | 115 | 0.025784 |
| sitosterol | 83 | 0.010530446 | 3,5-Dimethoxystilbene | 61 | 0.024598 |

**Table S7** Top 15 candidate targets in the C-T network according to degree and betweenness centrality.

| Protein Name | Degree | Betweenness Centrality | Protein Name | Degree | Betweenness Centrality |
| --- | --- | --- | --- | --- | --- |
| PTGS2 | 54 | 0.035328 | PTGS1 | 42 | 0.035343 |
| NCOA2 | 49 | 0.01164 | PTGS2 | 54 | 0.035328 |
| AR | 48 | 0.020706 | AR | 48 | 0.020706 |
| ESR1 | 46 | 0.004363 | PPARG | 23 | 0.015876 |
| PTGS1 | 42 | 0.035343 | ACHE | 18 | 0.013354 |
| RXRA | 31 | 0.009231 | NCOA2 | 49 | 0.01164 |
| GABRA1 | 29 | 0.003085 | TNF | 12 | 0.0116 |
| ESR2 | 28 | 0.001461 | PPARA | 12 | 0.01123 |
| PIM1 | 27 | 0.004446 | ADORA1 | 10 | 0.010569 |
| HSP90A | 27 | 0.001984 | SCN5A | 22 | 0.010239 |
| PRSS1 | 26 | 9.40E-04 | SHMT1 | 9 | 0.010117 |
| PGR | 25 | 4.98E-04 | ATP1A2 | 18 | 0.009873 |
| ATP1A1 | 24 | 0.005754 | XDH | 10 | 0.009636 |
| AHR | 24 | 0.004933 | RXRA | 31 | 0.009231 |
| PPARG | 23 | 0.015876 | IL1B | 8 | 0.009034 |
